# Gold Nanobipyramids for Near-Infrared Fluorescence-Enhanced Imaging and Treatment of Triple-Negative Breast Cancer

**DOI:** 10.3390/cancers15143693

**Published:** 2023-07-20

**Authors:** Ioannis G. Theodorou, Fotios Mpekris, Paris Papagiorgis, Myrofora Panagi, Maria Kalli, Louiza Potamiti, Kyriacos Kyriacou, Grigorios Itskos, Triantafyllos Stylianopoulos

**Affiliations:** 1 Cancer Biophysics Laboratory, Department of Mechanical and Manufacturing Engineering, University of Cyprus, Nicosia 1678, Cyprus; 2 Experimental Condensed Matter Physics Laboratory, Department of Physics, University of Cyprus, Nicosia 1678, Cyprus; 3 Department of Cancer Genetics, Therapeutics and Ultrastructural Pathology, The Cyprus Institute of Neurology and Genetics, Nicosia 2371, Cyprus

**Keywords:** nanoparticles, fluorescence enhancement, bioimaging, theranostic, targeting, in vivo study

## Abstract

**Simple Summary:**

Gold nanoparticles have gained significant attention in the field of cancer research and treatment due to their unique properties and potential applications to medical imaging, targeted drug delivery, and combined use for cancer diagnosis and therapy. In this study, we developed novel gold nanoparticles with a bipyramidal shape, which have been functionalized to improve fluorescence imaging in the near-infrared biological window and facilitate the targeted delivery of chemotherapy to triple-negative breast tumors. We successfully tested these nanoparticle formulations in both in vitro and preclinical tumor models. Their ability to be combined with a wide range of fluorophores, therapeutics, and targeting agents demonstrates their clinical potential against various cancer types.

**Abstract:**

There is an imminent need for novel strategies for the diagnosis and treatment of aggressive triple-negative breast cancer (TNBC). Cell-targeted multifunctional nanomaterials hold great potential, as they can combine precise early-stage diagnosis with local therapeutic delivery to specific cell types. In this study, we used mesoporous silica (MS)-coated gold nanobipyramids (MS-AuNBPs) for fluorescence imaging in the near-infrared (NIR) biological window, along with targeted TNBC treatment. Our MS-AuNBPs, acting partly as light amplification components, allow considerable metal-enhanced fluorescence for a NIR dye conjugated to their surfaces compared to the free dye. Fluorescence analysis confirms a significant increase in the dye’s modified quantum yield, indicating that MS-AuNBPs can considerably increase the brightness of low-quantum-yield NIR dyes. Meanwhile, we tested the chemotherapeutic efficacy of MS-AuNBPs in TNBC following the loading of doxorubicin within the MS pores and functionalization to target folate receptor alpha (FRα)-positive cells. We show that functionalized particles target FRα-positive cells with significant specificity and have a higher potency than free doxorubicin. Finally, we demonstrate that FRα-targeted particles induce stronger antitumor effects and prolong overall survival compared to the clinically applied non-targeted nanotherapy, Doxil. Together with their excellent biocompatibility measured in vitro, this study shows that MS-AuNBPs are promising tools to detect and treat TNBCs.

## 1. Introduction

The treatment of triple-negative breast cancer (TNBC) relies on non-targeted chemotherapy as the only treatment option, and although many patients initially respond well to this treatment, in most cases, the patients relapse with distant metastases [1]. Cell-targeted multifunctional nanomaterials hold great promise as new tools in TNBC management, providing effective platforms able to combine precise early-stage diagnosis with therapeutic delivery directly to tumors to increase drug potency and efficacy [2,3,4]. Nanoparticle (NP) functionalization is a widely investigated technique that allows for NP conjugation to targeting ligands, with presumed inherent abilities to selectively bind to specific cell types. This active targeting approach has been proposed to enhance the preferential “retention” of nanoparticles in tumors compared to the passive targeting conferred by the enhanced permeability and retention effect (EPR) [5]. For TNBC, a number of potential antigen targets have been suggested, and their clinical utility is currently under investigation [6]. Among these, folate receptor alpha (FRα) is highly expressed in non-mucinous tumors of epithelial origin, including ovarian, breast, and lung cancers. The specific overexpression of FRα in TNBC [7], along with its low coordinate expression in normal tissue, makes FRα an attractive target for directed nanomaterials.

Such targeted nanostructures may offer new possibilities for sensitive tumor detection with the concurrent application of the multimodal treatment of TNBC cells (i.e., “theranostic” applications). Among the diagnostic options available, fluorescence labeling and imaging have attracted widespread attention in clinical practice due to their significant potential in non-invasive disease prognosis, diagnosis, and therapy. However, a plateau in the technology has been reached because of the high light absorption by tissues in the visible wavelength range. Recently, fluorophores emitting in the near-infrared (NIR) windows (NIR-I: 650–900 nm and NIR-II: 1.0–1.7 μm) have received particular interest [8,9,10], because the low absorption of light by water and hemoglobin allows high transparency for tissue imaging [11]. Lower autofluorescence from organic molecules in this wavelength range also offers negligible background signals [12], while reduced photon scattering could allow imaging with high tissue penetration depths [13]. However, developing bright NIR fluorophores that are both photostable and biocompatible is still proving to be extremely challenging, hindering the further applicability of this technology. To overcome these obstacles, efforts have recently focused on metal-enhanced fluorescence (MEF), an optical process in which the near-field interaction of fluorophores with metallic nanoparticles can, under specific conditions, produce large fluorescence enhancements [14,15,16,17,18]. This light amplification may be exploited to considerably increase detection sensitivity, therefore improving the performance of fluorescence-based technologies [19]. The presence of metallic nanostructures in the vicinity of fluorophores could also stabilize them against photobleaching [20], further enhancing their use in bioimaging applications. Therefore, effective and scalable platforms for NIR fluorescence enhancement are highly desirable and could pave the way for novel high-performance diagnostic devices [21,22].

To date, although some platforms for fluorescence enhancement in the biological NIR window have been reported [23,24,25,26,27], these mainly focused on nanostructured surfaces or nanoparticles immobilized on solid substrates, like gold (Au) or silver (Ag) nanostructured arrays fabricated by nanosphere lithography, or Au nanostar (AuNS) self-assembled monolayers [28]. Such approaches allow for good control of the nanoparticle–fluorophore distance, and the interparticle distance may be tuned to optimize the magnitude of MEF, but this geometry limits the range of practical applications [10,29,30,31,32]. There have been only few examples of NIR fluorescence enhancement with colloidal nanomaterials. For instance, we recently examined the mechanism of MEF from colloidal AuNSs with controlled morphologies and tunable plasmonic responses [10], but their capabilities for in vitro or in vivo imaging were not further investigated. An emerging particle morphology with unexplored potential to significantly contribute to this field is the Au nanobipyramid (AuNBP). AuNBPs are elongated AuNPs with two sharp tips, tunable sizes, and NIR-tunable localized surface plasmon resonance (LSPR) [33]. Their high monodispersity, compared to other anisotropic NPs like Au nanorods (AuNRs) or AuNSs, affords lower inhomogeneous spectral broadening of their LSPR peaks, while their local electric field enhancements (one of the main factors driving MEF) are several times larger than those of AuNRs due to their sharp edges [34,35]. Finite-difference time-domain calculations have also shown that AuNBPs exhibit larger extinction cross-sections than AuNRs at similar LSPR wavelengths, making them more favorable for optical applications, including MEF [33].

Overall, gold nanoparticles have gained significant attention in the field of cancer research and treatment (Table 1) due to their unique properties and potential applications. The use of gold nanoparticles in cancer includes the following applications [36,37,38,39]: (i) Imaging: Gold nanoparticles can be used as contrast agents in various imaging techniques, such as computed tomography (CT), magnetic resonance imaging (MRI), and photoacoustic imaging. Their ability to absorb and scatter light makes them highly visible in imaging, allowing for enhanced tumor detection and visualization. (ii) Targeted drug delivery: Gold nanoparticles can be functionalized with various molecules, such as antibodies or targeting ligands, to specifically bind to cancer cells or tumor markers. These functionalized nanoparticles can carry and deliver therapeutic agents directly to cancer cells, improving drug efficacy while minimizing side effects on healthy tissues. (iii) Photothermal therapy (PTT): Gold nanoparticles exhibit a phenomenon known as the plasmonic effect, where they can efficiently convert absorbed light energy into heat. By selectively accumulating gold nanoparticles in tumor tissues and irradiating them with near-infrared light, the nanoparticles generate localized heat, leading to tumor cell destruction. (iv) Radiotherapy enhancement: Gold nanoparticles have the ability to enhance the effectiveness of radiation therapy. When exposed to ionizing radiation, gold nanoparticles can absorb and scatter X-rays, resulting in increased radiation dose deposition within the tumor. (v) Biosensing and diagnostics: Gold nanoparticles can be utilized in cancer diagnostics by detecting specific biomarkers or genetic material associated with cancer. Their unique optical properties enable the development of sensitive biosensors for early cancer detection and monitoring of the treatment response. (vi) Theranostics: Gold nanoparticles have the potential to combine therapeutic and diagnostic functionalities into a single platform. By integrating therapeutic agents and imaging capabilities within the same nanoparticle, theranostic systems enable personalized medicine, real-time monitoring of treatment efficacy, and targeted therapy.

Therefore, the aim of the present work was to engineer a new targeted multifunctional nanostructure that combines the NIR-MEF imaging of TNBC cells with local chemotherapeutic drug delivery. This nanostructure consists of the following: (i) A plasmonic AuNBP core that acts as a light amplification component for NIR dyes. In the future, this plasmonic core may also be utilized for the photothermal ablation of cancer cells [40], therefore conferring multimodal treatment capabilities to our nanostructure. (ii) A mesoporous silica (MS) coating around the AuNBPs, serving as a spacer for the controlled distance between the AuNBPs and the NIR fluorescent dyes, which is a prerequisite for a large fluorescence enhancement. Meanwhile, this MS layer, containing several channel-like nanopores, allows our nanostructures to serve as anticancer-drug carriers [2,41]. (iii) The surface labelling of the MS layer with the NIR fluorophore DyLight™ 800 (DL800), which is used to investigate its fluorescence enhancement by the AuNBP core and its potential in TNBC cell imaging, and (iv) surface functionalization of the nanostructures with folic acid (FA) for FRα targeting. Here, we show that dye conjugation to the AuNBPs allows considerable fluorescence enhancement of around 14 times compared to the free dye. Using time-resolved fluorescence analysis measurements, we also demonstrate a significant increase in the modified quantum yield for DL800 bound to AuNBPs, which illustrates that these particles can considerably increase the brightness of low-quantum-yield NIR dyes and therefore improve their possible performance in clinical applications. In the absence of drug loading, our nanostructures present excellent biocompatibility in vitro, indicating their suitability for such applications. Furthermore, in vitro fluorescence imaging and viability measurements show that FA-functionalized, doxorubicin-loaded particles target FRα-positive cells with significant specificity and reduce their viability more than free doxorubicin. Finally, using an in vivo TNBC model, we illustrate that our FRα-targeted particles induce antitumor effects and prolong overall survival in animals to a higher degree than a clinically applied non-targeted nanotherapy (Doxil).

**Table 1:** Applications of gold nanoparticles in cancer research.

Therapeutic Modality	Performance of Nanoparticles	References
Magnetic resonance imaging (MRI)	Use of superparamagnetic and paramagnetic nanoparticles for monitoring of various types of cancer	[42,43,44]
Computed tomography (CT)	Contrast agents to diagnose cancer	[45,46]
Targeted drug delivery	Surface functionalization of gold nanoparticles with the use of specific antibodies to provide targeted delivery	[47,48,49,50,51]
Photothermal therapy (PTT)	Treatment of primary and metastatic tumors through heating	[52,53,54,55,56]
Radiotherapy	Enhancement of the effectiveness of radiation therapy	[57,58]
Biosensing and diagnostics	Detection of specific biomarkers or genetic material associated with cancer	[59,60]
Theranostics	Combination of therapeutic and diagnostic functionalities into a single platform	[61,62]

## 2. Materials and Methods

The present section summarizes the main aspects of the experimental methods, with detailed descriptions provided in the Supplementary Material.

### 2.1. Synthesis of Mesoporous-Silica-Coated AuNBPs (MS-AuNBPs)

Gold nanobipyramids (AuNBPs) were synthesized via a modified seed-mediated growth method. To remove spherical gold nanoparticles (AuNPs) from the as-synthesized products [35], the samples were purified using Ag overgrowth, depletion-induced self-separation, and finally, chemical etching and removal of the overgrown Ag [35]. The purified AuNBPs were coated with a mesoporous silica (MS) shell via a modified Stöber method [31]. The resulting MS-coated products are identified as MS-AuNBPs. As confirmed by several transmission electron microscopy (TEM) images, very few cases of uncoated AuNBPs were observed; therefore, similar to previous studies [31,63], a purification step of uncoated AuNBPs from MS-AuNBPs was not performed.

### 2.2. Characterization of AuNBPs

As-prepared and purified AuNBPs and MS-AuNBPs were characterized by optical absorption spectroscopy using an Implen P330 nanophotometer. Transmission electron microscopy (TEM) was performed using a JEOL JEM-1010 with an accelerating voltage of 100 kV. The TEM images presented and the statistics on particle dimensions and MS coating were obtained by viewing several AuNBPs (n > 100 for each sample) from multiple areas of three samples prepared under identical conditions. Size distributions were measured using several TEM images and processed via ImageJ software version 1.53a (http://rsb.info.nih.gov/ij/).

### 2.3. Conjugation of Fluorophores and Targeting Agent to MS-AuNBPs

To functionalize the MS-AuNBPs with the commercially available NIR fluorophore DyLight™ 800 (DL800; Excitation 777 nm/Emission 790 nm) for imaging (MS-AuNBPs-DL), folic acid (FA) for TNBC cell targeting (MS-AuNBPs-FA), or both (MS-AuNBPs-DL-FA), their silica coating was first amino-modified using APTES. The formation of amino groups on the silica surface then allowed the covalent bonding of DL800-NHS ester molecules through the formation of amide bonds with the NHS ester. DL800 was selected as it has a very low quantum yield (estimated < 4%) and therefore represents a good candidate for investigating plasmon-enhanced fluorescence [15]. MS-AuNBPs were similarly functionalized with FA using an amine-reactive pegylated FA-NHS ester. MS-AuNBPs functionalized with DL800 only are identified as MS-AuNBPs-DL, those functionalized with FA only are identified as MS-AuNBPs-FA, and those functionalized with both as are identified as MS-AuNBPs-DL-FA.

The particle number concentration of the samples was estimated as previously described [35] from the Au mass concentration measured with Inductively Coupled Plasma–Optical Emission Spectroscopy (ICP-OES, Thermo Scientific, Cambridge, UK), and the average nanoparticle sizes were determined from TEM images. The particle number concentration of all samples was adjusted to be the same (1 × 10^11^ mL^−1^).

### 2.4. Doxorubicin Loading and Release Experiments

To load the anticancer drug doxorubicin (Dox) within the pores of the MS shell of the MS-AuNBPs or MS-AuNBPs-FA, the water-soluble hydrochloride salt of doxorubicin, Dox-HCl, was used. Dox loading was measured at 0.52 mg/mL. Dox-loaded particles are identified as MS-AuNBPs-Dox or MS-AuNBPs-Dox-FA, depending on the absence or presence of FA targeting. The release profiles of Dox from the MS-AuNBPs were measured at 37 °C in normal saline (NS) buffers, with their pH adjusted to 7.4 and 5.2 with NaOH or HCl, respectively [64]. The nanostructures were incubated at 50 μg/mL in terms of Dox concentration. Assuming that the solubility of Dox in NS is similar to that of PBS (0.5 mg/mL), the Dox release experiment was performed in sink conditions. To determine the amount of free Dox released from the incubated nanostructures over a time period of 0–24 h, aliquots were collected at selected time points (0, 1, 2, 3, 4, 6, 8, 10, 18, 24 h) and centrifuged at 14,000× *g* for 20 min to remove the nanostructures. The amount of free Dox in the supernatants was determined by optical absorption spectroscopy using the absorbance of Dox at 480 nm. Each experiment was repeated three times, and the results are given as the mean and standard deviation of the three repeats. Statistical analysis was performed with Microsoft Excel (Excel 2019, v16.0).

### 2.5. Optical Spectroscopy

Optical absorbance measurements were carried out on a Perkin Elmer Lambda 1050 spectrophotometer. Steady-state and time-resolved fluorescence experiments were performed on a 0.35 m FluoroLog FL3 Horiba Jobin Yvon spectrofluorimeter equipped with a visible PMT with a detection range of 250–850 nm. Sample excitation for the steady-state experiments was achieved via the monochromator-filtered output of a 450 W Xe lamp. Time-resolved fluorescence was measured using time-correlated single-photon counting (TCSPC), with the emission excited by a picosecond laser diode at 633 nm with a pulse width of ~50 ps.

### 2.6. Cell Culture and Cell Viability Assays

For in vitro experiments, the non-tumorigenic MCF10A human mammary epithelial cell line and the SKBR3 and MDA-MB-231 human mammary carcinoma cell lines were used. Cell viability was quantified using the Alamar blue, MTS, and LDH release assays. All cell viability experiments were performed in triplicate. Data are presented as mean ± standard error. Statistical analysis was performed using Student’s two-tailed *t*-tests (* *p* < 0.05; ** *p* < 0.01) with Microsoft Excel (Excel 2019, v16.0).

### 2.7. Cell Labeling and Imaging

Fluorescence cell imaging was performed following the labeling of cells cultured on glass coverslips. Images were acquired using an Olympus BX53 fluorescence microscope equipped with an Olympus XM10 IR Monochrome CCD camera.

### 2.8. Animal Tumor Models and Treatment Protocols

4T1 tumor models were generated by orthotopic implantation of 5 × 10^4^ 4T1 murine mammary carcinoma cells in 40 µL of serum-free medium into the mammary fat pad of 6–8-week-old female BALB/c mice. Our study consisted of 5 treatment groups: control, Doxil, MS-AuNBPs-FA, MS-AuNBPs-Dox, and MS-AuNBPs-Dox-FA. The untreated control mice were treated with saline throughout the study. For the rest of the groups, mice were treated with the nanotherapeutics (Doxil or AuNBP groups) once the tumor volume reached 250 mm^3^. The nanotherapeutics were administered via intravenous (i.v.) injection once a week for a total of 2 doses. When the average size in the control group reached a tumor burden of 1000 mm^3^, primary tumors were surgically removed, the abdominal surface and skin were sutured, and the mice were observed until time to death from metastasis. Animal survival was quantified based on time to death. Doxil, MS-AuNBPs-Dox-FA, and MS-AuNBPs-Dox were all administered at a concentration of 3 mg/kg in terms of doxorubicin content, and MS-AuNBPs-FA were administered at the same AuNBP particle number concentration as MS-AuNBPs-Dox-FA and MS-AuNBPs-Dox.

The planar dimensions (x, y) of the tumors were monitored every 2–3 days using a digital caliper, and tumor volume was estimated from the volume of an ellipsoid, assuming that its third dimension, z, was equal to $\sqrt{xy}$. Animal survival was quantified based on the time of death following the initiation of the treatment or the time to reach the maximum tumor burden (1200 mm^3^). All in vivo experiments were conducted in accordance with the animal welfare regulations and guidelines of the Republic of Cyprus and the European Union (European Directive 2010/63/EE and Cyprus Legislation for the protection and welfare of animals, Laws 1994–2013) under a license (No. CY/EXP/PR.L2/2018) acquired and approved by the Cyprus Veterinary Services committee, the Cypriot national authority responsible for monitoring all forms of animal research. Data are presented as mean ± standard error.

Groups were compared using Student’s two-tailed *t*-tests with Microsoft Excel (Excel 2019, v16.0). A *p* value less than or equal to 0.05 was considered statistically significant.

## 3. Results and Discussion

### 3.1. Synthesis of AuNBPs with Tunable Sizes and Optical Properties

**Figure 1:**
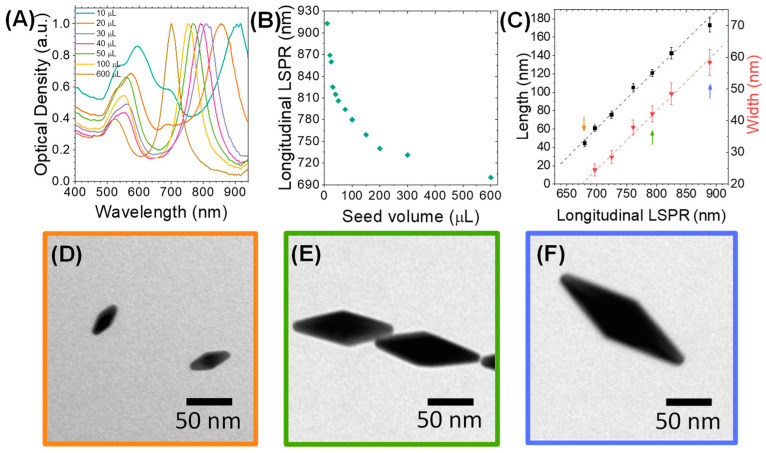
Morphological and optical properties of as-synthesized AuNBPs fabricated through seed-mediated wet chemical synthesis. (**A**) Normalized absorbance spectra of AuNBPs synthesized with different seed volumes added to the growth solution. (**B**) Longitudinal localized surface plasmon resonance (LSPR) peak position of AuNBPs grown with different seed volumes. (**C**) Dimensions of AuNBPs relative to the seed volume used for their growth. (**D**–**F**) Representative transmission electron microscopy (TEM) images of AuNBPs grown with three different seed volumes, corresponding to the points indicated with arrows of matching colors in (**C**). The TEM images presented and the statistics on particle dimensions were obtained by viewing several AuNBPs (n > 100 for each sample) from multiple areas of three samples prepared under identical conditions.

**Figure 2:**
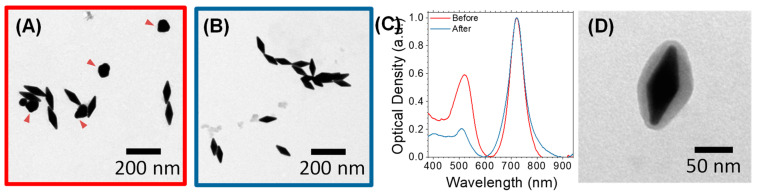
Purification process and mesoporous silica (MS) coating of AuNBPs. (**A**–**C**) Representative TEM images (**A**,**B**) and normalized absorbance spectra (**C**) of AuNBPs before (**A**) and after (**B**) purification to remove the roughly spherical Au nanoparticles grown as a by-product of the synthesis ((**A**), red arrowheads). (**D**) Representative TEM image of MS-coated AuNBPs (MS-AuNBPs).

First, gold nanobipyramids (AuNBPs) with tunable sizes and plasmonic responses in the NIR spectral region were synthesized via a modified seed-mediated growth method [35,65]. The optical properties of the AuNBPs were tuned in the NIR region by varying the seed volume added to the growth solution. Normalized optical absorbance spectra of the growth products are shown in Figure 1A, while Figure 2A shows a representative transmission electron microscopy (TEM) image of the sample grown using 300 μL of seeds. Each absorbance spectrum exhibited two peaks: a longer-wavelength peak attributed to the longitudinal LSPR resonance of the AuNBPs and a shorter-wavelength peak, which results from the contribution of both the transverse plasmon resonance of the AuNBPs and the plasmon resonance of roughly spherical Au nanoparticles that were grown as a by-product of the synthesis (Figure 2A, red arrowheads) [33]. Figure 1B shows that, by varying the seed volume, the longitudinal LSPR of the AuNBPs was tunable in a wide range of wavelengths in the NIR region, around 690-915 nm. This tunability of their optical properties was due to the different AuNBP dimensions, i.e., aspect ratios, obtained with each seed volume (Figure 1C–F) and could be indispensable for further optical applications, like MEF.

As evidenced from their absorbance spectra (Figure 1A) and TEM images (Figure 2A), the directly grown products contained a large number of roughly spherical Au nanoparticles. The AuNBP yield, determined using several representative TEM images, was around 53%. Therefore, the samples were purified using a previously described protocol based on Ag overgrowth, depletion-induced self-separation, and the ultimate chemical etching and removal of the overgrown Ag [35]. The purified AuNBPs (Figure 2B) had the same dimensions as the AuNBPs in the as-grown products, but their fraction in the purified samples increased to around 92%. This was also confirmed by the absorbance spectra of the purified samples (Figure 2C), which, after normalization, overlapped well with those of the initial samples at the longitudinal (longer-wavelength) peak but showed a significantly lower relative strength in the shorter-wavelength peak.

Since fluorescence enhancement, or quenching, is largely determined by the distance between fluorophores and metal surfaces, we used a mesoporous silica (MS) coating as a spacer around the AuNBPs. This MS coating, containing several channel-like pores, would also allow AuNBPs to serve as anticancer-drug carriers [3]. Silica is highly biocompatible [41], while its surface can be easily chemically modified to further functionalize the nanoparticles. MS-coated AuNBPs (MS-AuNBPs; Figure 2D) were prepared through a modified Stöber method [31], in which the Cetyltrimethylammonium Bromide concentration added allows for the control of the MS thickness. Several previous studies have already illustrated the distance dependence of MEF. For instance, the maximum fluorescence emission from PbS quantum dots coupled to AuNPs was obtained when the NPs were coated with a silica spacer of 10 nm [32]. Using AuNBPs, the maximum fluorescence intensity of the Cyanine7 (Cy7) dye was observed with ~14–17 nm spacers [63]. For our experiments, therefore, we selected to proceed with an MS coating of ~14 nm (Figure 2D), which is expected to be close to the optimal conditions for MEF.

### 3.2. Fluorescence Enhancement

**Figure 3:**
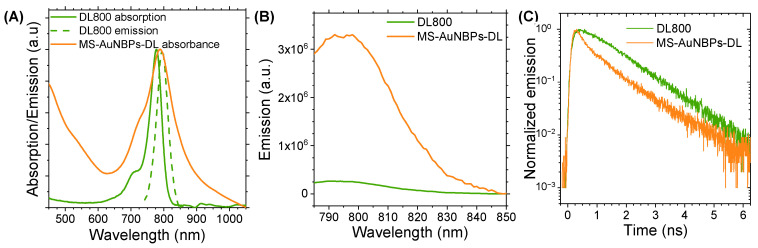
The DyLight™ 800 (DL800) near-infrared (NIR) dye conjugated to MS-AuNBPs exhibits significant fluorescence enhancement and fluorescence lifetime reduction compared to the free dye. (**A**) Normalized absorbance and emission spectra of DL800 in relation to the normalized absorbance spectrum of MS-AuNBPs. (**B**) Fluorescence emission spectra and (**C**) time-resolved decay curves of DL800 conjugated to MS-AuNBPs (MS-AuNBPs-DL) compared to equivalent amounts of free fluorophores.

One of the most important parameters that may influence the fluorescence enhancement factor of metal nanoparticles is the spectral overlap of their LSPR with the optical absorption and emission of the fluorophore. Therefore, for our fluorescence enhancement studies and the further development of our theranostic probe, we used AuNBPs with a longitudinal absorbance peak at 788 nm coated with a ~14 nm MS layer. Figure 3A shows the overlap between the LSPR peak of these MS-AuNBPs and the absorbance and fluorescence of the DL800 dye. Fluorescence enhancement was measured by quantifying the degree of dye conjugation to AuNBPs using optical absorption spectroscopy, as elaborated in the supplementary info text, and comparing the fluorescence intensity of the MS-AuNBPs-DL conjugate to that produced by an equal amount of free dye. The emission spectra of DL800 and MS-AuNBPs-DL, measured using an excitation wavelength of 768 nm, are shown in Figure 3B. The fluorescence enhancement factor (E_f_) for MS-AuNBPs-DL was calculated by comparing the integrated emission area of MS-AuNBPs-DL to that of DL800, normalized to the same amount of dye fluorophores as described above. We found a maximum E_f_ of around 13.6 times, showing that DL800 exhibited significant fluorescence enhancement when conjugated to MS-AuNBPs.

MEF is an intricate coupling process between fluorescent molecules and metal nanostructures, which can occur via (i) excitation enhancement, through which local enhancements of the electric field induced by the LSPR of metal nanoparticles can result in higher excitation rates of the fluorophores, and/or (ii) emission enhancement, which refers to the modification of the radiative and non-radiative decay rates of nearby fluorophores by metal nanoparticles, leading to a change in their lifetime and quantum yield [66,67]. Several parameters may influence the fluorescence enhancement factors of metal NPs, including particle size, shape, and the surrounding dielectric medium, as well as the particle arrangement geometry and the separation distance from fluorophores [29,67,68]. The E_f_ measured here is slightly higher than the 10.7-fold enhancement previously reported for Cy7 conjugated to AuNBPs. In our previous work, however, we reported up to 30 times fluorescence enhancement for DL800 bound to AuNSs [10]. This observation could be partly related to the local electromagnetic fields resulting from each particle morphology, which influences the excitation rate of the fluorophores. For AuNBPs, as well as AuNSs, the electric field enhancement is localized close to their sharp tips [69,70,71], with sharper tips producing larger enhancements [69,72]. Consequently, AuNS, with their multibranched morphology, may offer a large number of electric field hot spots per particle, ultimately contributing to a higher excitation enhancement. Nevertheless, the smaller overall size of AuNBPs, along with their elongated morphology, may provide distinct advantages for their tumor delivery in vivo by facilitating their transport across tumor vessel walls [5]. Therefore, the use of AuNBPs for fluorescence enhancement is a useful strategy for enhancing the detection sensitivity of low-quantum-yield fluorescent emitters and may prove a significant step forward in the in vivo application of MEF imaging.

The measured increases in fluorescence intensities can be attributed to the coupling between the fluorophores and AuNSs, but the mechanisms responsible for these increases are not immediately apparent. As noted, there could be different reasons for the increased emission, such as an increased radiative decay rate relative to the non-radiative decay rate or an effective increase in the excited-state population. To explore these effects and provide further insights into the mechanism of fluorescence enhancement from individual AuNBPs, we performed time-resolved fluorescence measurements. The fluorescence decay curves of free DL800, compared to dye conjugated to MS-AuNBPs, are shown in Figure 3C. Fluorescence lifetime data may be evaluated using a simple, single exponential (SE) model, suitable for describing the transient emission of single fluorophores in homogeneous environments, or a multi-exponential (ME) model that describes the fractional contribution of decay times from different components present in a sample mixture [73]. For our results, satisfactory fitting of the data was performed via an SE model in the case of the free dye and a model with two decay times for MS-AuNBPs-DL, as described in the Supplementary Material. The results are summarized in Table 2 and show that the average fluorescence lifetime of the dye was significantly reduced by a factor of ~26% when conjugated to the MS-AuNBPs.

The lifetime results were then analyzed within the framework of a well-established semi-empirical model for the plasmonic enhancement effects associated with fluorophores [74], as discussed in detail in the Supplementary Material, as well as our previous work [10,28,75]. The values for the excitation and emission enhancement factors are shown in Table 3 and demonstrate that the time-resolved fluorescence measurements allowed the semi-quantitative deconvolution of the two main mechanisms for the observed fluorescence enhancement. The results suggest that the main contribution to the fluorescence modification occurs due to an LSPR-induced increase in the radiative decay rates and a concomitant increase in the emission quantum yield of DL800 when conjugated to MS-AuNBPs. On the other hand, the excitation enhancement has a lower impact on the modification of the dye emission, with MS-AuNBPs supporting lower excitation enhancement factors compared to those previously measured with AuNSs. As noted earlier, this may be related to a larger concentration of electric field hot spots located close to the multiple spikes of AuNSs that induce a higher excitation enhancement in such nanostructures. The important result, though, is the significant increase in the modified quantum yield of DL800 bound to MS-AuNBPs, which indicates that such particles can considerably increase the brightness of low-quantum-yield NIR dyes, reaching those of the more efficient visible dye emitters. AuNBPs, which can be synthesized with controlled plasmonic responses (Figure 1A), could be spectrally coupled to several different NIR fluorophores in the future to explore the mechanism of MEF further and develop multiplexed bioimaging or biosensing platforms [21].

### 3.3. Targeted In Vitro Imaging

**Figure 4:**
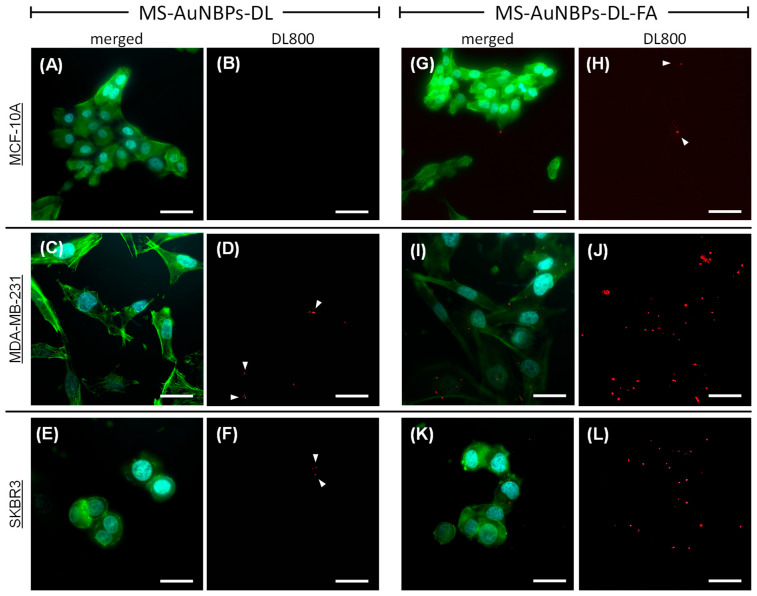
Folic acid-functionalized, DL-coated MS-AuNBPs (MS-AuNBPs-DL-FA) allow fluorescence imaging of folate receptor alpha (FRα)-positive cells with significant specificity. Fluorescence microscope images of MS-AuNBPs-DL in the absence (**A**–**F**) or presence (MS-AuNBPs-DL-FA; (**G**–**L**)) of FA functionalization, incubated with an FRα-negative epithelial cell line (MCF-10A; (**A**,**B**,**G**,**H**)), an FRα-positive triple-negative breast cancer (TNBC) cell line (MDA-MB-231; (**C**,**D**,**I**,**J**)), and an FRα-positive BC cell line (SKBR3; (**E**,**F**,**K**,**L**)) (cyan: DAPI; green: phalloidin; and red: DL800; (**A**,**C**,**E**,**G**,**I**,**K**): merged signals; (**B**,**D**,**F**,**H**,**J**): DL800 signal). White arrowheads indicate areas of weak NIR signal. Scale bar 20 μm.

Our optical spectroscopy experiments established the potential of MS-AuNBPs to provide a significant fluorescence enhancement of NIR dyes, which could be useful for cancer imaging applications with improved sensitivity and high contrast enhancement. Therefore, to test the applicability of our NPs as targeted imaging probes for tumor cells, we used them for in vitro cell labeling and fluorescence optical imaging. The NPs, in the absence (MS-AuNBPs-DL) or presence (MS-AuNBPs-DL-FA) of FA functionalization, were incubated with three different cell lines: MDA-MB-231, SKBR3, and MCF-10A. The MDA-MB-231 human mammary carcinoma cell line was used as a model for TNBC. As previously demonstrated, MDA-MB-231 cells, similar to all human TNBC cell lines tested, exhibit positive surface expression of the FRα protein [76,77]. As a comparison, we also tested the SKBR3 human mammary carcinoma cell line, which is HER2-positive but, similar to MDA-MB-231 cells, has a high expression of FRα [76,77]. As a control for normal cells, the MCF-10A human mammary epithelial cell line was employed, which expresses low levels of FRα [76]. The fluorescence images of MDA-MB-231, SKBR3, and MCF-10A cells are shown in Figure 4. The DAPI-stained nuclei of the cells are shown in cyan, the phalloidin-stained cell cytoskeleton is shown in green, and the NIR fluorescence of the DL800 fluorophore is shown in red (Figure 4A,C,E,G,I,K: merged signals; Figure 4B,D,F,H,J: DL800 signal). In the absence of FA conjugation, MCF-10A cells showed almost no DL800 signal (Figure 4A,B; NP area fraction 0.00%), while for both MDA-MB-231 (Figure 4C,D; NP area fraction 0.13%) and SKBR3 (Figure 4E,F; NP area fraction 0.03%) cells, there was a very weak NIR signal (Figure 4D,F: white arrowheads), indicating the low association of the MS-AuNBPs-DL with the cancer cells. In contrast, conjugation of the particles with FA led to a significantly increased amount of MS-AuNBPs-DL-FA associated with the FA-expressing cancer cells (Figure 4I–L). In the case of normal cells (Figure 4G), FA conjugation led to some particles being associated with the cells (Figure 4H, white arrowheads; NP area fraction 0.09%), but to a much lower degree compared to MDA-MB-231 cells (Figure 4J; NP area fraction 1.06%) and SKBR3 cells (Figure 4L; NP area fraction 0.49%), in accordance with their previously demonstrated levels of FRα expression [76]. These experiments qualitatively indicate that our FA-conjugated particles target FRα-positive cells with significant specificity and highlight the potential clinical utility of our MS-AuNBPs-DL-FA as imaging probes to specifically target cancer types with high FRα expression, like TNBC. Further experiments are underway in order to provide more quantitative data on FRα targeting and to test their potential for in vivo NIR imaging. In addition to diagnostic applications, the efficient targeting of our NPs to specific cell types could also allow concomitant local drug delivery to relevant cancer types. The FA targeting selected here was employed as a model to test their targeting possibilities, but the flexibility of the conjugation protocol used allows the versatility of our particles to a range of emerging targeting agents. For instance, MS-coated AuNSs loaded with ZnO nanoparticles and conjugated with the antibody to the Frizzled-7 (FZD-7) receptor have recently been illustrated as promising agents to treat triple-negative and drug-resistant breast cancers [3].

### 3.4. In Vitro Biocompatibility

**Figure 5:**
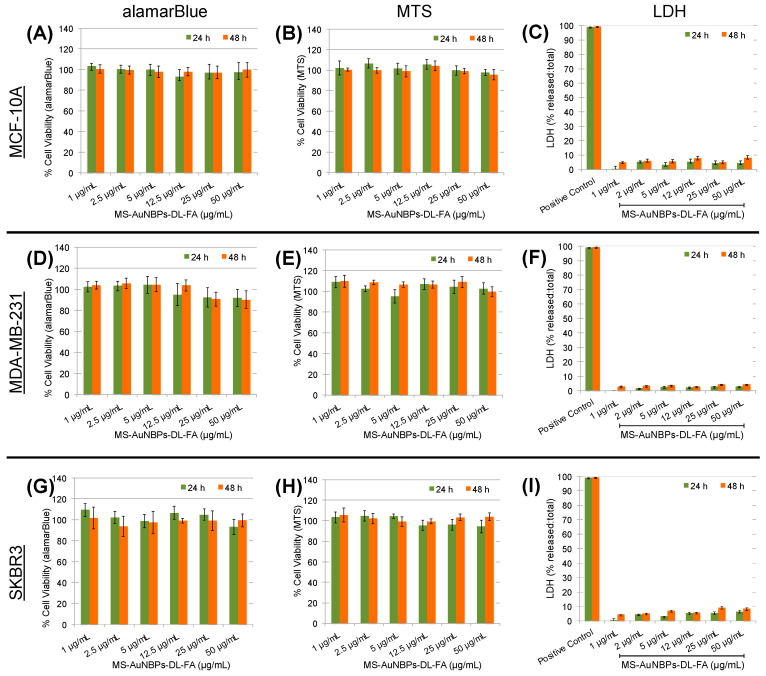
MS-AuNBPs-DL-FA present high biocompatibility in the absence of drug loading. Cell viability measured with the Alamar blue assay ((**A**,**D**,**G**); n = 3), MTT assay ((**B**,**E**,**H**); n = 3), and LDH release ((**C**,**F**,**I**); n = 3) following treatment of the MCF-10 (**A**–**C**), MDA-MB-231 (**D**–**F**), and SKBR3 (**G**–**I**) cell lines with 0–50 μg/mL MS-AuNBPs-DL-FA for 24 and 48 h. Data are presented as mean ± standard error. Statistical analysis was performed using Student’s two-tailed *t*-tests.

To test the applicability of MS-AuNBPs-DL-FA for the further development of in vivo imaging and therapeutic applications, we proceeded to test their in vitro biocompatibility in the absence of drug loading with both healthy and cancer cells. Like our fluorescence imaging experiments, the MCF-10A cell line, which is a widely used in vitro model for studying normal breast cell function, was employed as a control for normal cells [78]. All cell lines were treated with a dose range of 0–50 μg/mL MS-AuNBPs-DL-FA for 24 and 48 h (Figure 5). Biocompatibility was assessed using the Alamar blue assay (Figure 5A,D,G), as well as a tetrazolium dye assay (MTT; Figure 5B,E,H), which measures the metabolic activity of cells. As another indicator of MS-AuNBPs-DL-FA biocompatibility, we used the LDH release assay (Figure 5C,F,I), which quantifies cell membrane permeability. Compared to the non-treated controls, cell viability was not significantly changed for any of the cell lines under any exposure conditions. A slight reduction in viability was only observed with the Alamar blue assay in the case of MDA-MB-231 cells exposed to 25 and 50 μg/mL MS-AuNBPs-DL-FA (Figure 5D), but viability was still higher than 90%. In addition, no significant LDH release was measured in any of the experiments. Therefore, without drug loading, MS-AuNBPs-DL-FA exhibited negligible toxicity to the cell lines used at the studied doses, indicating the suitability of our designed particles for in vivo applications. Similarly, studies have previously reported negligible cytotoxicity for MS-coated AuNRs and AuNSs, in accordance with the high biocompatibilities of both the Au core and the silica coating [41,79].

### 3.5. Drug Loading and In Vitro Chemotherapeutic Potential

**Figure 6:**
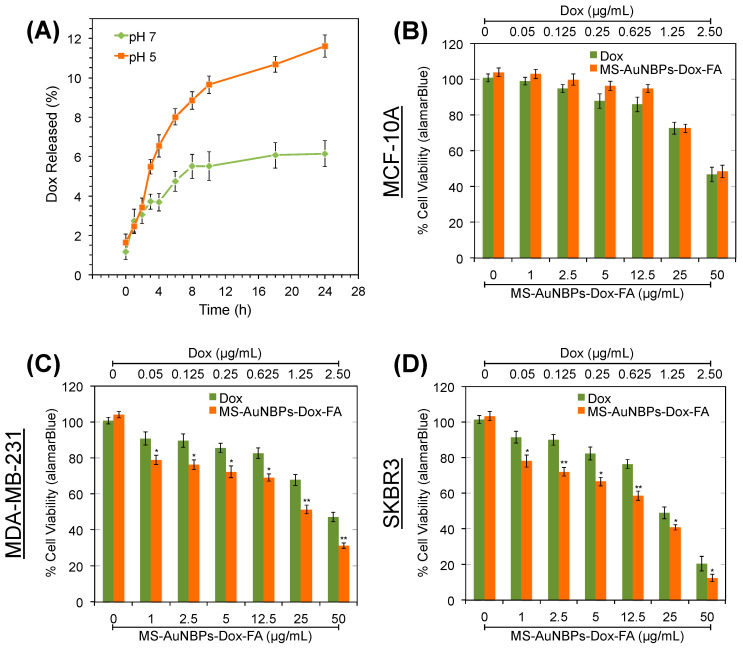
FRα-targeted MS-AuNBPs loaded with the anticancer drug doxorubicin (Dox) increase its potency compared to free Dox. (**A**) Release profile of Dox from MS-AuNBPs-Dox-FA measured at 37° in pH 7.4 and pH 5.2 buffers. Each experiment was repeated three times, and the results are given as the mean and standard deviation of the three repeats. (**B**–**D**) Cell viability measured with the Alamar blue assay (n = 3) following treatment of the MCF-10 (**B**), MDA-MB-231 (**C**), and SKBR3 (**D**) cell lines with 0–50 μg/mL MS-AuNBPs-Dox-FA and 0–2.5 μg/mL Dox for 24 h. Data are presented as mean ± standard error. Statistical analysis was performed using Student’s two-tailed *t*-tests (* *p* < 0.05; ** *p* < 0.01).

The potential of our MS-AuNBPs as anticancer-drug carriers was explored using doxorubicin (Dox). Dox-loaded particles are hereafter referred to as MS-AuNBPs-Dox or MS-AuNBPs-Dox-FA, depending on the absence or presence of FA targeting. The Dox loading was measured to be 0.52 mg/mL. The in vitro drug release profiles of MS-AuNBPs-Dox were measured at 37° in buffers with pH 7.4 and pH 5.2 to mimic extracellular and lysosomal pH, respectively (Figure 6A). At pH 7.4, drug release was slow and reached a plateau after 8 h. A faster rate of sustained Dox release was observed at pH 5.2, with around 12% of drug released measured after 24 h. Previous studies have also reported the pH-dependent release of Dox from MS [80,81], which could be due to the protonation of more surface silanols with the decrease in pH, leading to a decrease in the electrostatic interaction and dissociation of Dox from the silica surface. In the future, strategies like capping the silica channels with pH-responsive gatekeepers [80] or using laser irradiation to enhance Dox release [81] could further improve our control over Dox release from the MS-AuNBPs. Still, the pH dependence observed here suggests that drug release is expected to be relatively low extracellularly but higher in the acidic intracellular environment of breast cancer cells, which could subsequently induce cell death.

The chemotherapeutic potential of the MS-AuNBPs-Dox-FA was explored using the Alamar blue cell assay to quantify the viability of MDA-MB-231 and SKBR3 breast cancer cells and MCF-10A epithelial cells (Figure 6B–D). Cells were treated with a dose range of 0–50 μg/mL MS-AuNBPs-Dox-FA for 24 h, as well as equivalent amounts of free Dox, assuming a 10% release of Dox from MS-AuNBPs-Dox over 24 h (Figure 6A). Both free Dox and MS-AuNBPs-Dox-FA induced dose-dependent cytotoxicity, with SKBR3 cells showing the highest decrease in cell viability among the cell lines tested. In addition, for the cell lines with higher FRα expression (Figure 6C–D), the targeted NPs were significantly more toxic than free Dox. This observation may be related to the higher association of MS-AuNBPs-DL-FA with FRα-positive cells observed in our fluorescence imaging experiments (Figure 4). In accordance with these findings, we have previously seen that the in vitro anticancer potency of FZD-7-targeted AuNSs correlated with the level of FZD-7 expression by the cell lines tested [3]. Even in the absence of active targeting, previous work suggests that loading Dox into nano-drug carriers can increase drug uptake and accumulation within cells. For instance, flow cytometry experiments have shown that 5–10 times more Dox was internalized within A549 human lung cancer cells when it was loaded in MS-coated AuNRs compared to free Dox [81].

### 3.6. FA targeting Increases the In Vivo Antitumor Efficacy of Doxorubicin-Loaded Particles

**Figure 7:**
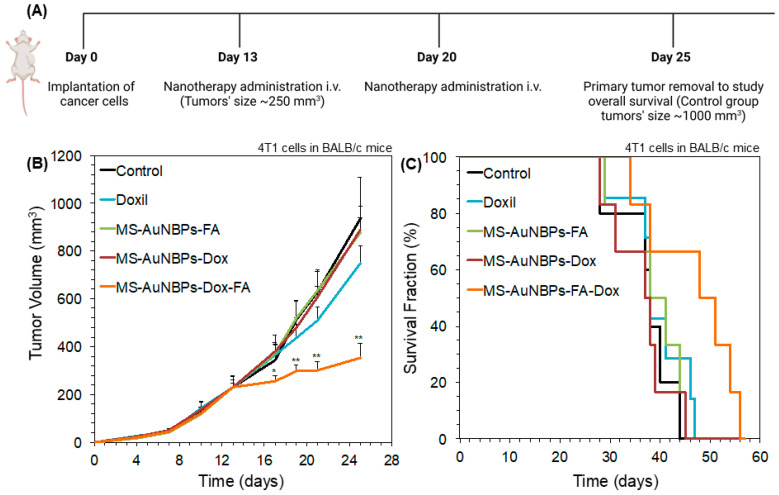
MS-AuNBPs-Dox-FA exert antitumor effects and prolong overall survival in a syngeneic, orthotopic TNBC mouse model to a higher degree than a clinically applied non-targeted nanotherapy (Doxil). (**A**) Schematic of the experimental protocol. Created with BioRender.com. Tumor volume growth rates (**B**) and animal survival (**C**) for 4T1 breast cancer murine tumors implanted in BALB/c female mice. Animals were treated with saline as a control, Doxil as a comparison with non-targeted nanotherapy, and MS-AuNBPs in the presence of FA functionalization (MS-AuNBPs-FA), Dox loading (MS-AuNBPs-Dox), or both (MS-AuNBPs-Dox-FA). Data are presented as mean ± standard error. Groups were compared using Student’s two-tailed *t*-tests. A *p* value less than or equal to 0.05 was considered statistically significant (*: statistical significance vs. control group; **: statistical significance vs. all other treatment groups).

Finally, to test the in vivo antitumor efficacy of our doxorubicin-loaded FA-conjugated particles (MS-AuNBPs-Dox-FA), we employed a syngeneic, orthotopic TNBC mouse model using the 4T1 murine mammary adenocarcinoma cell line. The 4T1 cell line overexpresses FRα, enabling the potential use of FA-functionalized vesicles for the targeted delivery of chemotherapeutic drugs to these cells [82,83]. Animals were treated with either saline as a control, Doxil as a comparison with a clinically applied but non-targeted nanotherapy, or MS-AuNBPs-Dox-FA (Figure 7A). To illustrate the efficacy of targeting, we also used a group of animals treated with doxorubicin-loaded particles not conjugated with FA (MS-AuNBPs-Dox). Finally, to preclude any effects from FA alone, we used FA-conjugated particles not loaded with doxorubicin (MS-AuNBPs-FA). The therapeutic efficacy of each agent was evaluated by measuring tumor growth (Figure 7B) and the overall survival of the treated animals (Figure 7C). We found that MS-AuNBPs-FA and MS-AuNBPs-Dox did not induce a significant delay in tumor growth compared to the untreated group. Interestingly, treatment with Doxil also induced only a marginal decrease in tumor size. In contrast, MS-AuNBPs-Dox-FA led to a significant delay in tumor growth, resulting in around 60% smaller tumors after 25 days. Similarly, MS-AuNBPs-FA, MS-AuNBPs-Dox, and Doxil had no effect on animal survival compared to the control. In contrast, MS-AuNBPs-Dox-FA significantly improved overall survival. These results illustrate the potential of our targeted nanoprobe to exert antitumor effects and prolong overall survival in vivo. These effects could be attributed to an increased accumulation of MS-AuNBPs-Dox-FA at the tumor site, which could possibly be due to the combined effects of passive targeting stemming from their size and their active molecular targeting, ultimately resulting in a higher local delivery of doxorubicin. Further investigation will allow us to determine the in vivo imaging capabilities of our particles and quantify their tumor accumulation. Still, our current findings suggest that MS-AuNBPs-Dox-FA are a promising platform for the treatment of TNBC while possessing imaging capabilities.

## 4. Conclusions

Our study shows the potential clinical utility of our targeted multifunctional nanostructure based on MS-AuNBPs for the concomitant imaging and treatment of breast cancer. Our fluorescence spectroscopy studies provide important insights into the mechanism of MEF from MS-AuNBPs, which can be used to further guide particle design for a large contrast enhancement, enabling the development of ultrasensitive MEF biodetection technologies. In particular, the ability to systematically tune the plasmonic responses of AuNBPs could allow spectral coupling to several different NIR fluorophores, paving the way to multiplexed bioimaging or biosensing applications. Furthermore, the high flexibility of our nanostructure to several therapeutic modalities and targeting agents allows for modifications to further improve its potency and efficacy. For instance, in parallel to localized drug delivery, the AuNBP core could also be used as a photothermal therapy mediator for the multimodal treatment of aggressive subtypes of breast cancers [84]. Moreover, apart from doxorubicin, emerging strategies that could be further investigated include loading the MS nanopores with zinc in the form of either ZnO nanoparticles or Zn^2+^ ions, which have shown promise in the treatment of drug-resistant cancers [2,3,4].

## Figures and Tables

**Table 2:** Bi-exponential analysis of time-resolved decays of DL800 before and after conjugation to MS-AuNBPs, showing the weighting fractions (*a*_1_ and *a*_2_), the observed lifetimes (τ_1_ and τ_2_), and the intensity-weighted average lifetime (τ).

Sample	*a*_1_	*a*_2_	τ_1_ (ns)	τ_2_ (ns)	τ (ns)
DL800	-	-	1.33	-	1.33
MS-AuNBPs-DL	0.47	0.53	1.19	0.36	0.98

**Table 3:** Calculated values of modified quantum yield (Q_m_), total enhancement factor (E_f_), emission enhancement factor (E_em_), and excitation enhancement factor (E_ex_) of DL800 (emission 794 nm) conjugated to MS-AuNBPs.

Sample	Q_m_	E_f_	E_em_	E_ex_
MS-AuNBPs-DL	0.29	13.6	7.3	1.9

## Data Availability

Data can be retrieved from corresponding author upon reasonable request.

## Supplementary Materials

The following supporting information can be downloaded at: https://www.mdpi.com/article/10.3390/cancers15143693/s1, Detailed descriptions of the experimental methods are provided in the Supplementary Material. References [31,35,63,64,65,73,74,85,86,87,88] are cited in the supplementary materials.
