# Supplementary material for: Gold Nanobipyramids for Near-Infrared Fluorescence-Enhanced Imaging and Treatment of Triple-Negative Breast Cancer
Source: Cancers (Basel). 2023 Jul 20;15(14):3693. doi: 10.3390/cancers15143693 (PMC10378199; doi:10.3390/cancers15143693)
Supplement: Supplementary file 1 [file cancers-15-03693-s001.zip › cancers-2476938-supplementary.pdf]

Supplementary Material

Gold Nanobipyramids for Near-Infrared Fluorescence Enhanced Imaging and Treatment of Triple Negative Breast Cancer

1. Materials

Gold chloride trihydrate ($HAuCl_4 \cdot 3H_2O$), Silver nitrate ($AgNO_3$), trisodium citrate dihydrate, silver nitrate ($AgNO_3$), Sodium borohydride ($NaBH_4$), L-ascorbic acid (AA), Hexadecyltrimethylammonium bromide (CTAB), Cetyltrimethylammonium chloride (CTAC) solution (25% w/w in H_2O), Tetraethyl orthosilicate (TEOS), (3-Aminopropyl)triethoxysilane (APTES), Hydrochloric acid (HCl, ≥37%), Nitric acid (HNO_3), sodium hydroxide (NaOH), N,N-Dimethylformamide (DMF; anhydrous, 99.8%), Ammonium hydroxide solution (NH_3, 28.0-30.0%), Hydrogen peroxide solution (H_2O_2; 30% w/w in H_2O) and doxorubicin hydrochloride (Dox-HCl) were purchased from Sigma-Aldrich, UK. DyLight™ 800 (DL800)-N-Hydroxysuccinimide (NHS) Ester was purchased from Life Technologies Limited, UK. Folic acid-Polyethylene glycol-NHS Ester (FA-PEG-NHS; 3400 Da) was purchased from Nanocs Inc. (Boston, MA, USA). Pegylated liposomal doxorubicin (Doxil; 2 mg/ml) was purchased from Janssen Pharmaceutica (Beerse, Belgium). De-ionized (DI) water purified using the Millipore Mili-Q gradient system (>18.2 MΩ) was used for all the experiments.

2. Methods

2.1 Synthesis of Gold Nanobipyramids

Gold Nanobipyramids (AuNBPs) were synthesized *via* a modified seed-mediated growth method. As previously discussed, the morphology of the Au nanoparticle product resulting from such synthetic protocols, critically depends on the seed morphology [1]. When single crystal seeds are used as a precursor, the growth process leads to the formation of AuNRs, whereas polycrystalline seeds result to the formation of AuNBPs [1]. Here, we used a previously proposed protocol based on cetyltrimethylammonium chloride (CTAC)-stabilized seeds, which has been suggested to lead to more reactive species, as a result of the lower affinity of CTAC to the Au surface [2]. The seeds were synthesized through the reduction of $HAuCl_4$ in the presence of CTAC, following fast addition of a reducing agent solution [2]. Briefly, the seed solution was prepared by mixing 0.2 mL of 10 mM $HAuCl_4 \cdot 3H_2O$, 7.8 mL of 50

mM CTAC and 72 μL of 250 mM HNO_3 at 20°C. Then, 50 μL of ice-cold freshly-prepared 100 mM $NaBH_4$/100 mM NaOH solution were quickly under vigorous stirring (~1200 rpm). The mixture turned from light yellow to faint red indicating the formation of the gold seeds. The mixture was stirred for five minutes to eliminate any hydrogen formed through the decomposition of $NaBH_4$, followed by the addition of 1.6 mL of 10 mM trisodium citrate dihydrate. The seed vials were then tightly sealed and aged in a water bath set at 80°C for 60 minutes, during which the solutions turned to a brighter red colour, indicating a slight increase in size. Aging the gold seeds at 80°C further ensured an increase in the polycrystallinity and size of the final seeds [2]. Finally, the vials were removed from the water bath and stored at room temperature (RT). For AuNBP growth [3], a growth solution was prepared through sequential addition of 1 mL of 0.01 M $HAuCl_4 \cdot 3H_2O$, 0.2 mL of 0.01 M $AgNO_3$, 0.4 mL of 1 M HCl and 0.16 mL of 0.1 M AA into 20 mL of a 0.1 M aqueous CTAB solution. Then, 10-600 μL of the seed solution were added into the growth solution and gently mixed by inversion for 15 s. The reaction solution was kept undisturbed overnight at RT. To characterize as-synthesized nanoparticles, the AuNBPs were washed x3 with DI water by centrifugation at 11000 rpm for 10 min at RT.

2.2 Purification of Au NBPs

To remove spherical gold nanoparticles (AuNPs) from the as-synthesized products [4], 20 mL of as-grown solutions were centrifuged at 11000 rpm for 20 min. The precipitates were collected and redispersed into 15 mL of a 0.08 M aqueous CTAC solution. Then, 4 mL of a 0.01 M $AgNO_3$ and 1.2 mL of a 0.1 M AA solutions were added to the CTAC solutions and gently mixed by inversion. The solutions were placed in a water bath set at 65°C for 4 h to allow for Ag overgrowth on the AuNBPs and AuNPs. The resulting Au/Ag products were centrifuged at 5000 rpm for 10 min, and the precipitates were redispersed into 15 mL of 0.05 M CTAB solutions, and left undisturbed overnight at RT. Then, the supernatants were carefully removed, and the remaining precipitates were redispersed in 10 mL water. Next, 495 μL of 25% ammonia and 450 μL of 30% H_2O_2 were added to these solutions. The mixtures were left undisturbed for 3 h, after which their color changed from grey-green to brown, and white AgCl precipitated at the bottom of the container. The supernatant was carefully removed, centrifuged at 10000 rpm for 10 min and washed x3 with DI water, and redispersed in 6 mL of DI water for further use.

2.3 Synthesis of mesoporous silica-coated AuNBPs (MS-AuNBPs)

The purified AuNBPs were coated with a mesoporous silica (MS) shell *via* a modified Stöber method [5], The purified AuNBP solution (6 mL) was sonicated for 10 min and then 720 µL of 0.01 M CTAB were added. The solution was kept on an orbital shaker for 24 h at RT to allow for the CTAB bilayer to adsorb on the AuNBP surface. Then, 120 µL of 0.1 M NaOH were quickly added to the AuNBPs, and the solutions were stirred on an orbital shaker for 30 min. Next, 720 µL of a 2% TEOS solution in ethanol were slowly added and allowed to react with the AuNBPs for 24 h at 45°C. The resulting products (MS-AuNBPs) were centrifuged at 10000 rpm for 15 min, washed x1 with DI water and x3 with ethanol, and redispersed in 6 mL ethanol.

2.4 Characterization of AuNBPs

As-prepared and purified AuNBPs, and MS-AuNBPs were characterized by optical absorption spectroscopy, using an Implen P330 nanophotometer. Extinction spectra were collected using glass cuvettes from Hellma® Analytics, and a 100% transmittance (T) baseline correction using DI water or ethanol, to account for water/cuvette absorption, was applied to the data. Transmission electron microscopy (TEM) was performed using a JEOL JEM-1010 with an accelerating voltage of 100 kV. Size distributions were measured using several TEM images and processed *via* ImageJ software (http://rsb.info.nih.gov/ij/).

2.5 Conjugation of fluorophores and targeting agent to MS-AuNBPs

The NIR fluorophore investigated in this study was DyLight™ 800 (DL800), which is commercially available. DL800 was selected because it is more hydrophobic, photostable and bright, but less pH-sensitive, compared to other commercially available dyes emitting in the NIR region. These features make it more suitable for cell and tissue imaging applications [6]. The NHS ester-activated derivative of the fluorophore was obtained, which is amine-reactive, allowing easy conjugation to amino groups. Therefore, the surface of the MS-AuNBPs was first modified with amino groups. Briefly, 54 µL of 28-30%, ammonium hydroxide were added to the MS-AuNBP solutions, and stirred for 30 min at RT. Then, 300 µL of 10% APTES in ethanol were added and the solutions were stirred for 3 h at 35°C and 1 h at 65°C. The amino-modified MS-AuNBPs were washed by centrifugation x3 with ethanol. To conjugate

the DL800 fluorophore, the DL800-NHS ester was dissolved in DMF at 0.1 mg/mL, and 108 μL were added to 6 mL of the amino-modified MS-AuNBPs. The mixtures were allowed to react for 10 h at RT. The fluorophore-conjugated MS-AuNBPs-DL800 were washed x3 with phosphate buffered saline (PBS; pH 7.4) by centrifugation and stored in PBS at 4°C for further use. The supernatants of each sample from both these washes with PBS were combined, and centrifuged again at 10000 rpm for 20 min to remove any residual MS-AuNBPs. The absorption spectra of each supernatant were measured to determine dye loading of the MS-AuNBPs. All absorption spectra were collected from 3 independent samples in each case. All the sample containers were covered with aluminum foil throughout the experiments to prevent the dye from photobleaching.

To functionalize the amino-modified MS-AuNBPs with folic acid (FA), we used FA-PEG-NHS, an amine reactive pegylated folic acid. FA-PEG-NHS was dissolved in DMF at 0.1 mg/mL, and 360 μL were added to 6 mL of the amino-modified MS-AuNBPs. The mixtures were allowed to react for 10 h at RT, followed by the same washing procedure as described for DL800 conjugation.

MS-AuNBPs functionalized with *DL800 only* are labelled as MS-AuNBPs-DL, those functionalized *only with FA* as MS-AuNBPs-FA, while those functionalized with *both* as MS-AuNBPs-DL-FA.

2.6 Doxorubicin loading and release experiments

To load the anticancer drug doxorubicin (Dox) within the pores of the MS shell of the MS-AuNBPs or MS-AuNBPs-FA, the water-soluble hydrochloride salt of doxorubicin, Dox-HCl, was dissolved in PBS. The particles were re-dispersed in PBS at Dox-HCl concentrations of 1 mg/mL. The solutions were stirred for 24 h at RT and the Dox-loaded particles were collected by centrifugation and washed several times by centrifugation with PBS to remove excess Dox. The supernatants from these washes were collected and the absorbance of Dox at 480 nm was used to determine the loading efficiency, by comparing to the absorbance of the initial Dox-HCl solution. The Dox loading was found to be 0.52 mg/mL. Dox-loaded particles are labelled as MS-AuNBPs-Dox or MS-AuNBPs-Dox-FA, depending on the absence or presence of FA-targeting.

The release profiles of Dox from the MS-AuNBPs were measured at 37°C in two buffers, with pH 7.4 and pH 5.2, respectively [7]. To determine the amount of free

Dox released from the incubated nanostructures over the time course of 0-24 h, aliquots were collected at selected time points and centrifuged at 14 000g for 20 min to remove the nanostructures. The amount of free Dox in the supernatants was determined by optical absorption spectroscopy using the absorbance of Dox at 480 nm. Each experiment was repeated three times and the results are given as the mean and standard deviation of the three repeats.

2.7 Optical Spectroscopy

To investigate the mechanism of fluorescence enhancement, AuNBP extinction spectra were collected using a Perkin Elmer Lamda 1050 spectrophotometer, equipped with a three-detector module covering the 300 - 3000 nm spectral range. Steady state photoluminescence (PL) and photoluminescence excitation (PLE) experiments were performed on a 0.35 m FluoroLog FL3 Horiba Jobin Yvon spectrofluorimeter, equipped with a TBX-04 visible PMT with a detection range of 250 - 850 nm. Sample excitation was achieved *via* the monochromator-filtered output of a 450 W ozone-free Xe lamp.

Time-resolved photoluminescence (TRPL) was acquired using the monochromator-based time-correlated single-photon counting (TCSPC) method on the same setup used for the steady-state PL experiments. PL was excited using a laser diode at 633 nm with a pulse width of ~50 ps operating at a repetition rate of 1 MHz. The excitation beam had a diameter of ~0.5 mm, and PL decay was collected while monitoring the PL emission peak with a spectral bandwidth of 16 nm.

Fluorescence lifetime data can be evaluated using single exponential (SE) models, which are suitable for single fluorophores in homogeneous environments, or multiexponential (ME) models, which describe the fractional contribution of decay times from different components present in a sample mixture [8]. For our results, satisfactory fitting of the data required a SE model in the case of free dye, and a model with two decay times for MS-AuNBPs-DL:

$$I(t) = a_1 e^{-\frac{t-t_o}{\tau_1}} + a_2 e^{-\frac{t-t_o}{\tau_2}} \qquad \text{(S1)}$$

In the second case, the average decay time τ was calculated from the two decay times τ_1 and τ_2, using the weighting a_1 and a_2 for each of the decay components:

$$\tau = \frac{\sum_1^n \alpha_i \tau_i^2}{\sum_1^n \alpha_i \tau_i} \qquad (S2)$$

The results of fitting our data to this model are summarized in Table 1, and show that the average fluorescence lifetime was significantly reduced for DL800 conjugated to MS-AuNBPs. Recent literature in MEF has established a widely accepted semi-empirical model for the plasmonic enhancement effects associated with fluorophores [9] . For isolated fluorophores, in the absence of metal nanoparticles or any quenching interactions, their emission can be described in terms of their quantum yield (Q_0) and lifetime (τ_0). Q_0 is defined as the ratio of radiative relaxation rate (Γ_0) to the total relaxation rate (Γ_0+k_{nr}), where k_{nr} is the non-radiative decay rate:

$$Q_0 = \frac{\Gamma_0}{\Gamma_0+k_{nr}} \qquad (S3)$$

The lifetime is given by the inverse of the total decay rate:

$$\tau_0 = \frac{1}{\Gamma_0+k_{nr}} \qquad (S4)$$

For fluorophores close to metal nanoparticles, enhancement of the near field can increase the energy absorbed by the fluorophore. Additionally, electromagnetic coupling between the fluorophore and the LSPR of the nanoparticles, increases the radiative decay rate of the fluorophore by a rate Γ_m. Therefore, the modified quantum yield (Q_m) and lifetime (τ_m) are:

$$Q_m = \frac{\Gamma_m+\Gamma_0}{\Gamma_m+\Gamma_0+\Gamma_{m,abs}+k_{nr}} \qquad (S5)$$

$$\tau_m = \frac{1}{\Gamma_m+\Gamma_0+\Gamma_{m,abs}+k_{nr}} \qquad (S6)$$

$\Gamma_{m,abs}$ is the additional non-radiative decay rate, whose effectiveness falls off rapidly with separation distance, and thus the modified quantum yield tends back to its original value at far enough separation. As the separation distance becomes very small

(<5 nm), the fluorophore is quenched by the metal nanoparticle, until complete quenching occurs. In our experiments, the spacing between fluorophores and AuNBPs provided by the MS spacer (~14 nm), prevents a significant increase in the non-radiative decay rate. Therefore, the non-radiative decay rates for DL800 before and after conjugation to AuNBPs can be considered equal, meaning that the term $\Gamma_{m,abs}$ can be neglected.

Using the measured fluorescence lifetimes of DL800, and assuming an unmodified quantum yield of 4% for DL800 [10] as its exact value is not known, we calculated the modified quantum yield for MS-AuNBPs-DL through equations (3)-(6). Thus, the emission enhancement factor, defined as $E_{em} = Q_m/Q_0$, could be derived. Finally, the excitation enhancement factor was calculated using: $E_{ex} = E_f/E_{em}$.

2.8 Cell culture

The non-tumorigenic MCF10A human mammary epithelial cell line was obtained by the Karmanos Cancer Institute (Detroit, MI, USA) and was cultured in Dulbecco's Modified Eagle's Medium Nutrient Mixture-12 medium (DMEM/F12, Gibco), supplemented with 5% Horse Serum (HS), 1% antibiotics, 10 mg/ml insulin, 20 ng/ml epidermal growth factor (EGF), 100 ng/ml cholera toxin (CT), and 500 ng/ml hydrocortisone (HC).

The SKBR3 and MDA-MB-231 human mammary carcinoma, and the 4T1 *murine* mammary carcinoma cell lines were purchased from ATCC® (Manassas, VA, USA). SKBR3 cells were cultured in McCoy's 5a Modified Medium (Gibco), supplemented with 10% fetal bovine serum (FBS) and 1% antibiotics. MDA-MB-231 cells were maintained in DMEM medium (Gibco), supplemented with 10% FBS and 1% antibiotics. 4T1 cells were maintained in Roswell Park Memorial Institute medium (RPMI, biosera) supplemented with 10% FBS and 1% antibiotics.

All cells were incubated at 37°C in a humidified incubator with 5% (v/v) CO_2. Cells were detached from culture with trypsin (0.05%) and ethylenediaminetetraacetic acid (EDTA, 0.02%) and resuspended in media for passaging to wells.

2.9 Cell viability assays

Alamar blue assay: Alamar blue detects the impairment of cellular metabolism. MCF10A, SKBR3 and MDA-MB-231 cells were grown in 96-well plates (5000

cells/well). Before cell treatment, each NP formulation was diluted in complete medium and ultrasonicated for 10 min at RT. Immediately after sonication, cells were exposed to the NP formulations at concentrations 0-50 μg/mL for 24 h. The Alamar blue assay was used according to the manufacturer's protocol. The spectrophotometric absorbance was measured at two wavelengths: 570 nm (reduced form of Alamar Blue) and 600 nm (oxidized form of Alamar Blue) using a RT-2100C Rayto microplate reader. The mean absorbance reading from the wells without the particles was used as the control (100% viability).

MTS assay: The MTS (3-(4,5-dimethylthiazol-2-yl)-5-(3-carboxymethoxyphenyl)-2-(4-sulfophenyl)-2H–tetrazolium) assay was also used to quantify metabolic activity, to correlate to the results of the Alamar blue assay. Following the same NP treatment as described for the Alamar blue assay, cells were washed with complete medium and incubated with fresh complete medium (100 μL) containing 10 μL of MTS reagent (Abcam, UK). Cells were incubated at 37°C for 1-2 h and the optical density at 490 nm was measured to determine intracellular NADH levels. Viability was determined from the optical density as a percentage of control cells.

LDH release: Lactate dehydrogenase (LDH) release was used to quantify cellular membrane integrity as another parameter of NP biocompatibility. Following 24 h of NP treatment, 10 μL of cell medium were transferred to 96-well plates and mixed with 100 μL of LDH reagent (Abcam, UK) to measure the levels of released (extracellular) LDH. Cells were then lysed using the kit's cell-lysis solution (in the original incubation medium) and 10 μL of cell medium was transferred to 96-well plates and mixed with 100 μL of LDH reagent to measure total (intracellular plus extracellular) LDH. Optical density at 490 nm was measured after 5-10 mines following addition of the LDH reagent, with cell viability quantified as the optical density ratio of released LDH to total LDH.

All cell viability experiments were performed in triplicate. Data are presented as mean ± standard error. Statistical analysis was performed using Student's two-tailed t-tests (* $p < 0.05$; ** $p < 0.01$).

2.10 Cell labeling and imaging

MCF10A, SKBR3 and MDA-MB-231 cells were plated at a density of 10 000 cells per well on glass coverslips coated with 0.1% gelatin. Twenty-four (24) hours

later, cells were fixed with 4% PFA for 20 min and they were then permeabilized using a buffer containing 0.1% Triton X-100 and 2 mg/ml BSA in PBS. Cells were then incubated with 25 μg/mL of NPs in PBS for 2 h in the dark on an orbital shaker at RT. Then, the coverslips were rinsed several times with PBS to remove unbound NPs. Cells were finally double stained with Rhodamine phalloidin (Biotium) and 4′,6-Diamidino-2-Phenylindole (DAPI; Roche) in the dark [11], according to the manufacturer's protocol. Phalloidin is a widely-used fungal toxin known to bind filamentous actin, and was used in order to stain the cell cytoskeleton, while DAPI is a fluorescent stain that binds strongly to adenine–thymine-rich regions in DNA, and was used to stain the cell nucleus. The coverslips were mounted on microscope slides using the ProLong™ Gold Antifade Mountant (Invitrogen). Cell images were acquired using an Olympus BX53 fluorescence microscope, equipped with an Olympus XM10 IR Monochrome CCD camera, and the following filters from Chroma Technology: 49000 ET-DAPI for DAPI, 49004 ET-CY3/TRITC for Phalloidin and 49037 ET-Li-Cor for DL800.

References

1. Liu, M.; Guyot-Sionnest, P. Mechanism of silver(I)-assisted growth of gold nanorods and bipyramids. *The journal of physical chemistry. B* **2005**, *109*, 22192-22200, doi:10.1021/jp054808n.
2. Chateau, D.; Liotta, A.; Vadcard, F.; Navarro, J.R.; Chaput, F.; Lerme, J.; Lerouge, F.; Parola, S. From gold nanobipyramids to nanojavelins for a precise tuning of the plasmon resonance to the infrared wavelengths: experimental and theoretical aspects. *Nanoscale* **2015**, *7*, 1934-1943, doi:10.1039/c4nr06323f.
3. Niu, C.; Song, Q.; He, G.; Na, N.; Ouyang, J. Near-Infrared-Fluorescent Probes for Bioapplications Based on Silica-Coated Gold Nanobipyramids with Distance-Dependent Plasmon-Enhanced Fluorescence. *Analytical chemistry* **2016**, *88*, 11062-11069, doi:10.1021/acs.analchem.6b03034.
4. Li, Q.; Zhuo, X.; Li, S.; Ruan, Q.; Xu, Q.-H.; Wang, J. Production of Monodisperse Gold Nanobipyramids with Number Percentages Approaching 100% and Evaluation of Their Plasmonic Properties. *Advanced Optical Materials* **2015**, *3*, 801-812.
5. Abadeer, N.S.; Brennan, M.R.; Wilson, W.L.; Murphy, C.J. Distance and plasmon wavelength dependent fluorescence of molecules bound to silica-coated gold nanorods. *ACS nano* **2014**, *8*, 8392-8406, doi:10.1021/nn502887j.
6. Panchuk-Voloshina, N.; Haugland, R.P.; Bishop-Stewart, J.; Bhalgat, M.K.; Millard, P.J.; Mao, F.; Leung, W.Y.; Haugland, R.P. Alexa dyes, a series of new fluorescent dyes that yield exceptionally bright, photostable conjugates. *The journal of histochemistry and cytochemistry : official journal of the Histochemistry Society* **1999**, *47*, 1179-1188, doi:10.1177/002215549904700910.
7. Theodorou, I.G.; Muller, K.H.; Chen, S.; Goode, A.E.; Yufit, V.; Ryan, M.P.; Porter, A.E. Silver Nanowire Particle Reactivity with Human Monocyte-Derived Macrophage Cells: Intracellular Availability of Silver Governs Their Cytotoxicity. *ACS biomaterials science & engineering* **2017**, *3*, 2336-2347, doi:10.1021/acsbiomaterials.7b00479.

8. Lakowicz, J.R.; Masters, B.R. Principles of fluorescence spectroscopy. *Journal of Biomedical Optics* **2008**, *13*, 9901.
9. Dragan, A.I.; Geddes, C.D. Metal-enhanced fluorescence: The role of quantum yield, Q0, in enhanced fluorescence. *Applied Physics Letters* **2012**, *100*, 093115.
10. Tynan, C.J.; Clarke, D.T.; Coles, B.C.; Rolfe, D.J.; Martin-Fernandez, M.L.; Webb, S.E. Multicolour single molecule imaging in cells with near infra-red dyes. *PloS one* **2012**, *7*, e36265, doi:10.1371/journal.pone.0036265.
11. Louca, M.; Stylianou, A.; Minia, A.; Pliaka, V.; Alexopoulos, L.G.; Gkretsi, V.; Stylianopoulos, T. Ras suppressor-1 (RSU-1) promotes cell invasion in aggressive glioma cells and inhibits it in non-aggressive cells through STAT6 phospho-regulation. *Scientific reports* **2019**, *9*, 7782, doi:10.1038/s41598-019-44200-8.
